# Current Dietary Practices in Hereditary Fructose Intolerance: Results from a National Survey Across 15 Italian Metabolic Centres

**DOI:** 10.3390/nu18162609

**Published:** 2026-08-10

**Authors:** Giulia Bruni, Juri Zuvadelli, Silvia Maria Bernabei, Cristina Bonfanti, Barbara Borsani, Alice Dianin, Annalisa Finizii, Aurora Favaro, Sara Giorda, Giorgia Gugelmo, Maria Paola Ierardi, Chiara Luciano, Marta Muscarella, Chiara Pancaldi, Sara Parolisi, Veronica Perico, Valentina Pierattini, Cinzia Pistolesi, Lidia Pontillo, Roberta Pretese, Angela Ira Pozzoli, Sara Quattrini, Alice Re Dionigi, Maria Giulia Regazzi, Alice Rossi, Simona Salera, Martina Tosi

**Affiliations:** 1 Dietetic Unit, Meyer Children University Hospital IRCCS, 50139 Florence, Italy; 2 Clinical Department of Paediatrics, ASST Santi Paolo e Carlo, San Paolo Hospital, University of Milan, 20142 Milan, Italy; juri.zuvadelli@asst-santipaolocarlo.it (J.Z.);; 3 Nutritional Rehabilitation Unit, SITRA, Bambino Gesù Children’s Hospital IRCCS, 00165 Rome, Italy; 4 Department of Pediatrics, Fondazione IRCCS San Gerardo Hospital, University Milano Bicocca, 20900 Monza, Italy; 5 Department of Pediatrics, Vittore Buzzi Children’s Hospital, University of Milan, 20154 Milan, Italymartina.tosi@unimi.it (M.T.); 6 Regional Center for Screening, Diagnosis, and Treatment of Inherited Metabolic Diseases, Pediatrics C Unit, University Hospital of Verona, 37126 Verona, Italy; 7 Department of Pediatrics, Metabolic Diseases, University of Turin, 10124 Turin, Italy; 8 Division of Metabolic Diseases, Department of Medicine, University Hospital of Padua, 35128 Padua, Italy; 9 Nutritional Center, Giannina Gaslini Pediatric Institute IRCCS, 16147 Genoa, Italy; 10 Clinical Dietietics Unit, IRCCS AOUBO, 40138 Bologna, Italy; 11 UOSD Metabolic Diseases, AORN Santobono-Pausilipon, 80129 Naples, Italy; 12 Dietetic Unit, Careggi University Hospital, 50134 Florence, Italy; 13 Pediatrics and Neonatology Unit, Regional Referral Clinical Center for Inborn Errors of Metabolism, Guglielmo da Saliceto Hospital, 29121 Piacenza, Italy; 14 Department of Pediatrics, Polytechnic University of Marche, G. Salesi Children’s Hospital, 60123 Ancona, Italy; 15 Regional Clinical Center for Expanded Newborn Screening, Fondazione IRCCS Ca’ Granda Ospedale Maggiore Policlinico, 20122 Milan, Italy

**Keywords:** Hereditary Fructose Intolerance, fructose, sweeteners, vegetables, fibre, multivitamin supplementation, dietitian, survey

## Abstract

**Background/Objectives**: Hereditary fructose intolerance (HFI) is a rare autosomal recessive metabolic disorder caused by variants in the *ALDOB* gene, resulting in aldolase B deficiency. Symptoms occur after the ingestion of fructose, sucrose, or sorbitol, leading to metabolic toxicity. Management is exclusively dietary, but clinical practice remains highly variable. This survey aimed to investigate current Italian dietary management practices for patients with HFI. **Methods**: A cross-sectional centre-level survey was conducted using a 29-item questionnaire distributed to metabolic dietitians within the Italian Society for the Study of Inherited Metabolic Diseases network between April and May 2026. **Results**: Data from 27 dietitians across 15 Italian inherited metabolic disease centres were analysed. Over half of the centres (53.3%) provided care for both paediatric and adult patients, and all centres (100%) supplied structured educational materials for the fructose-, sucrose-, and sorbitol-restricted diet. Marked heterogeneity was observed in dietary practices, particularly in the classification of sugars and sweeteners, recommendations for cereal-based products, and fibre supplementation strategies. Cereal-based dietary guidance was provided by 10/15 centres (66.7%), ranging from no specific recommendations (5/15, 33.3%) to a preference for refined grains (7/15, 46.7%) or fibre-based restrictions (2/15, 13.3%). With regard to sugars and sweeteners, consensus was observed only for maltitol and sorbitol, which were classified as prohibited by all centres (15/15, 100%). Mannitol and isomalt were prohibited in 14/15 centres (93.3%). All other compounds showed variable recommendations across centres, including xylitol (prohibited 46.7%, permitted 40.0%), erythritol (permitted 60.0%), and sucralose (prohibited 33.3%, permitted 20.0%, restricted 20.0%, unspecified 26.7%). Vitamin C (100%) and folic acid (86.6%) were the most commonly prescribed supplements. One-third (33%) of the centres required the use of fibre supplements or osmotic laxatives to manage chronic constipation, with wide variability in the specific type of fibre recommended. **Conclusions**: These findings highlight substantial heterogeneity in dietary management of HFI across specialised Italian centres and support the development of evidence-based, standardised nutritional guidelines to improve the consistency of care, nutritional adequacy, and patient outcomes.

## 1. Introduction

Hereditary fructose intolerance (HFI; OMIM #229600) is a rare, life-threatening autosomal recessive metabolic disorder caused by pathogenic variants in the *ALDOB* gene (located on chromosome 9q22.3) [1]. This genetic defect results in a deficiency of the enzyme aldolase B (fructose-1-phosphate aldolase), which is primarily expressed in the liver, renal cortex, and small intestinal mucosa [2,3]. The estimated prevalence of HFI ranges from 1:10,000 to 1:20,000 live births worldwide, although underdiagnosis remains a significant concern due to clinical heterogeneity and milder phenotypes [4,5].

Under physiological conditions, dietary fructose undergoes initial phosphorylation by ketohexokinase (fructokinase) to form fructose-1-phosphate (F-1-P). In healthy individuals, aldolase B subsequently cleaves F-1-P into the trioses dihydroxyacetone phosphate and glyceraldehyde, which directly enter the glycolytic and gluconeogenic pathways to support cellular energy production and systemic glucose homeostasis [6]. In patients with HFI, the catalytic deficiency of aldolase B leads to the rapid intracellular accumulation of F-1-P, particularly within hepatocytes and renal tubular cells, triggering severe cellular toxicity and metabolic dysregulation. The high concentration of intracellular F-1-P competitively inhibits glycogen phosphorylase and fructose-1,6-bisphosphatase, thereby halting both glycogenolysis and gluconeogenesis, which clinically manifests as acute postprandial hypoglycaemia. Concurrently, the downstream stimulation of pyruvate kinase accelerates the accumulation of lactate, pyruvate, and alanine, leading to metabolic acidosis [7].

Clinical manifestations typically emerge during infancy, coinciding with the introduction of fructose-, sucrose-, or sorbitol-containing foods, such as fruits, vegetables, and commercial infant formulas, into the diet [6]. Fructose derived from dietary sources, as well as endogenous fructose generated through sorbitol metabolism, contributes to F-1-P accumulation [5]. Acute exposure to these sugars can provoke immediate and severe symptoms, including nausea, vomiting, abdominal pain, protracted diarrhoea, lethargy, hypokalaemia, and renal tubular dysfunction, which may progress to acute liver failure [1,8]. Conversely, chronic, low-dose exposure frequently results in insidious clinical features, including failure to thrive, malnutrition, hyperuricemia, hepatic steatosis, and progressive, irreversible damage to both the liver and kidneys [1,8]. While systemic metabolic consequences are well-documented, secondary gastrointestinal manifestations, such as altered gut motility and chronic abdominal discomfort, are frequently reported by patients, though their underlying pathophysiological mechanisms remain incompletely understood [2].

Because gastrointestinal and general metabolic symptoms overlap significantly with common paediatric and adult disorders, such as dietary fructose malabsorption, irritable bowel syndrome (IBS), or non-alcoholic fatty liver disease (NAFLD), HFI is frequently misdiagnosed or recognized only after a significant diagnostic delay [9,10]. Interestingly, many individuals with undiagnosed HFI survive into adulthood by developing a subconscious, lifelong aversion to sweet-tasting foods, a protective behavioural mechanism that partially shields them from severe, acute complications. Historically, diagnosis relied on invasive fructose tolerance tests or liver biopsies to assess enzyme activity; however, molecular analysis of the *ALDOB* gene via targeted sequencing currently represents the non-invasive gold standard for definitive diagnosis [2].

To date, there is no causative pharmacological treatment for HFI. Disease management relies exclusively on a strict, lifelong dietary restriction of fructose, sucrose, and sorbitol (FSS), together with the systematic supplementation of potentially deficient micronutrients, particularly water-soluble vitamins [11]. However, maintaining strict dietary compliance is challenging. FSSs are ubiquitous not only in natural foods but also as hidden texturizers, sweetening agents, and excipients in commercial processed foods and pharmaceutical formulations, posing a constant risk of accidental exposure. Furthermore, even in patients who strictly adhere to an FSS-restricted diet, endogenous fructose production via the polyol pathway (wherein glucose is converted to sorbitol and subsequently to fructose) can contribute to persistent, subclinical metabolic alterations, including long-term hepatic steatosis [2,5].

Designing and implementing effective nutritional protocols remains challenging. There is an absence of universally accepted, evidence-based dietary thresholds for daily fructose intake, and nutritional recommendations vary substantially between metabolic centres internationally [11]. Because the management of such a restrictive diet requires complex behavioural adaptations, this heterogeneity in nutritional advice can directly impact long-term metabolic control, nutritional adequacy, and the overall quality of life of both patients and their families.

Despite the critical role of nutritional therapy, comprehensive data regarding real-world dietary management practices in HFI remain extremely scarce, particularly within European cohorts. In day-to-day clinical practice, metabolic dietitians are the primary professionals responsible for translating theoretical biochemical restrictions into sustainable, everyday nutritional plans. Yet, their specific clinical protocols, monitoring strategies, and supplementation criteria have never been systematically documented.

To our knowledge, this study represents the first Italian multicentre investigation aimed at mapping the heterogeneity of dietary management practices adopted for patients with HFI. By conducting a survey among specialized metabolic dietitians, this study aimed to investigate current real-world practices, focusing on nutritional restriction limits, micronutrient supplementation strategies, and long-term clinical and nutritional monitoring protocols.

## 2. Materials and Methods

### 2.1. Survey Development

A specific cross-sectional centre-level survey was developed using a structured questionnaire to systematically investigate current dietary management practices for HFI reported by Italian specialised metabolic centres. The survey included 29 items, comprising 27 closed-ended multiple-choice questions (some allowing multiple selections) and 2 open-ended questions designed to capture descriptive clinical protocols (Supplementary Material File S1). The questionnaire was developed for content validity and clinical relevance by an expert panel of metabolic dietitians with extensive experience in the nutritional management of inherited metabolic diseases (IMDs). The final version was approved by the Dietetics and Nutrition Working Group of the Italian Society for the Study of Inherited Metabolic Diseases (SIMMESN).

### 2.2. Study Population and Distribution

The target population consisted of Italian metabolic dietitians affiliated with SIMMESN who provide specialised dietary management for patients with IMDs. The survey was distributed to SIMMESN-affiliated metabolic centres identified as providing care for patients with IMDs (*n* = 20).

The questionnaire was addressed to metabolic dietitians working within eligible centres and was distributed electronically in Italian via official SIMMESN mailing lists. The survey was hosted on the secure Google Forms platform and remained open for data collection for a two-month period between April and May 2026. Participation was voluntary, and all respondents provided electronic informed consent before accessing the survey items. While the identities of the participants were known during data collection to ensure single-centre consensus representation, all data were fully anonymised prior to statistical analysis.

**Figure 1:**
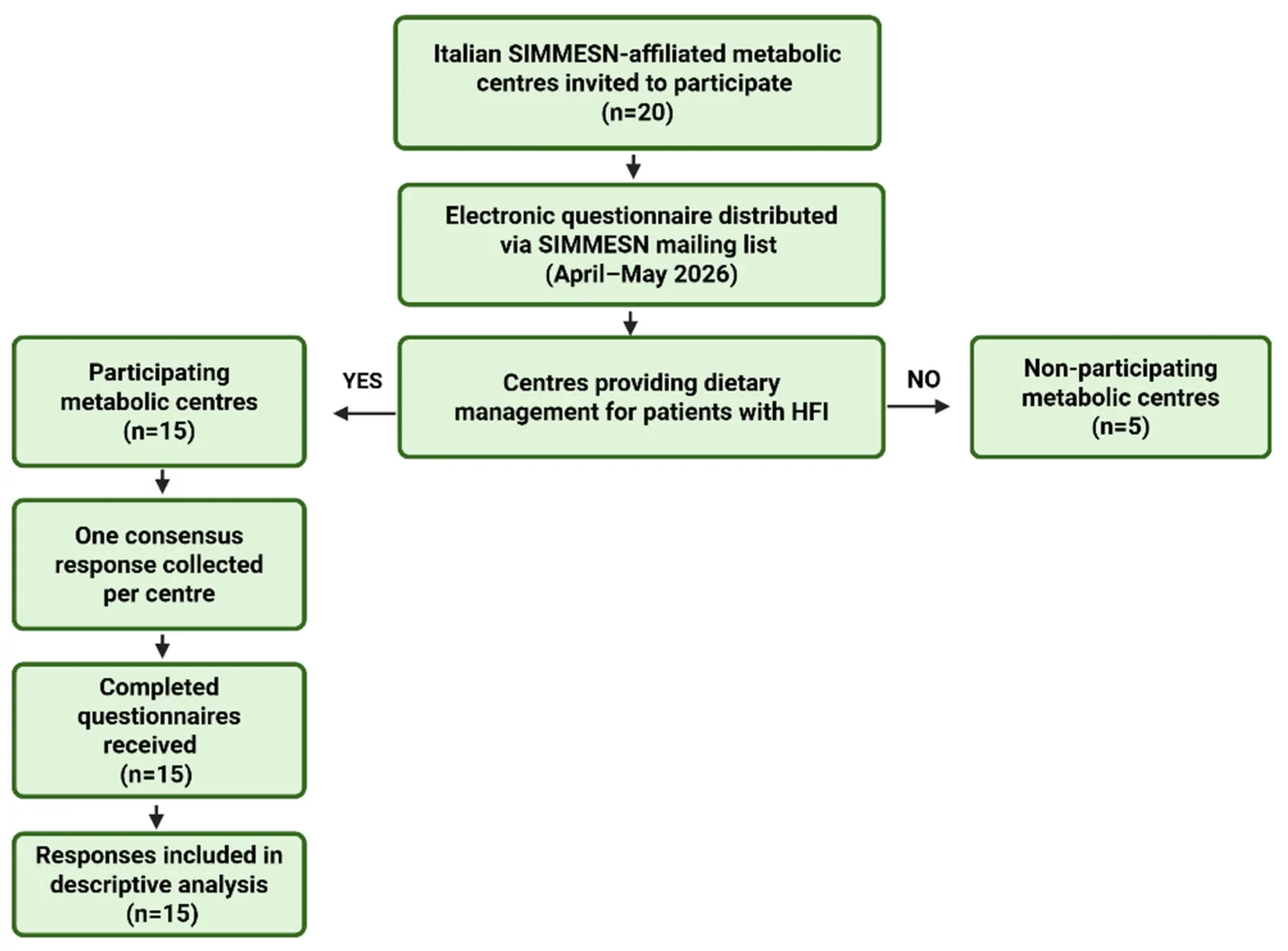
Overview of the survey design and data collection process. Abbreviations: SIMMESN: Italian Society for the Study of Inherited Metabolic Diseases; HFI: Hereditary Fructose Intolerance.

The flow diagram illustrating survey development, identification of eligible SIMMESN-affiliated IMDs centres, questionnaire distribution, centre participation, and inclusion of one consensus response per centre in the final analysis is reported in Figure 1.

### 2.3. Data Quality and Survey Domains

To ensure consistency of reporting and avoid over-representation of individual centres, only one comprehensive response per clinical centre was included in the final analysis. When multiple dietitians worked within the same institution, participants were instructed to coordinate and submit a single consensus response reflecting their centre’s overall dietary management practice.

The questionnaire was structured into four thematic domains:

- Centre Characteristics: classification of the clinical service (paediatric, adult, or mixed/transition care).
- Dietary Management Protocols: clinical approaches regarding the introduction or exclusion of specific vegetables and the quantitative or qualitative criteria used to define acceptable fructose thresholds in foods.
- Alternative Carbohydrates and Sweeteners: current clinical practices regarding the safety, inclusion, or restriction of alternative sugars, polyols, and high-intensity sweeteners.
- Supplementation and Monitoring: protocols regarding micronutrient supplementation (e.g., water-soluble vitamins) and routine nutritional follow-up.

Ethical approval was not required for this survey of healthcare professionals, as no patient-level data or identifiable personal information were collected.

### 2.4. Statistical Analysis

Statistical analysis was primarily descriptive. Quantitative and categorical variables derived from multiple-choice questions are expressed as absolute numbers and relative percentages of the total number of responding centres. For the qualitative data obtained from the two open-ended questions, a descriptive thematic analysis was performed. Two investigators independently reviewed the narrative responses to identify recurring patterns, which were subsequently categorised into consensus themes and summarised narratively to complement the quantitative findings. Given the exploratory descriptive design and the centre-level unit of analysis, no inferential statistical comparisons were planned.

## 3. Results

### 3.1. Centres’ Characteristics

A total of 27 metabolic dietitians completed the survey, representing 15 distinct Italian IMD centres, corresponding to 75% of Italian metabolic centres. In accordance with the study protocol, data were analysed on an institutional basis using one consensus response per centre. The geographical distribution of the participating centres showed a higher density in Northern Italy (*n* = 10/15, 66.7%), followed by Central Italy (*n* = 4/15, 26.7%) and Southern Italy (*n* = 1/15, 6.7%). Regarding the clinical setting of participating centres, the majority provided mixed care for both paediatric and adult populations (*n* = 8/15, 53.3%), while 5/15 centres (33.3%) managed exclusively paediatric patients, and 2/15 (13.3%) cared exclusively for adult patients.

### 3.2. Dietary Protocols

Universal consensus was observed regarding initial patient education: all responding centres (15/15, 100%) provided patients with specific, institution-developed written educational materials outlining permitted and prohibited foods for the FSS-restricted diet. Conversely, protocols regarding vegetable introduction revealed minor variations: 1/15 centres (6.7%) excluded all types of vegetables, while 14/15 (93.3%) applied a selective approach based on lists of allowed and excluded vegetables. Among the 14 centres adopting a selective approach, a high degree of homogeneity was observed in the quantitative fructose thresholds used to define vegetable acceptability. Specifically, 5/14 centres (35.7%) utilised a strict cut-off of less than 0.5 g of fructose per 100 g of raw product, whereas 9/14 (64.3%) applied a threshold of less than 1 g per 100 g. Furthermore, regarding portion control, 8/14 centres (57.1%) provided patients with indicative maximum portion sizes for allowed vegetables, while 6/14 centres (42.9%) did not specify any portion limits.

### 3.3. Classification of Sugars, Polyols, and Alternative Sweeteners

In contrast to vegetable management, the classification and clinical use of alternative carbohydrates, polyols, and high-intensity sweeteners revealed substantial heterogeneity across centres. Most compounds were variably categorised as prohibited, permitted, or permitted only in strictly controlled amounts, with several items omitted entirely from local dietary protocols. Sorbitol and maltitol were classified as forbidden in all centres (15/15, 100%), closely followed by mannitol and isomalt, which were prohibited in 14/15 centres (93.3%). In contrast, high rates of acceptability were reported for saccharin (14/15, 93.3%), aspartame (13/15, 86.7%), acesulfame K (12/15, 80.0%), and maltose (11/15, 73.3%). For all other investigated sugars and sweeteners, classifications varied widely among centres, as detailed in Table 1.

### 3.4. Natural Flavourings and Complex Cereal-Based Product Guidelines

The inclusion of commercial foods containing natural flavourings showed a lack of complete consensus among centres. Foods containing natural flavourings were permitted in 11/15 centres (73.3%), provided that the ingredient list explicitly excluded any other prohibited sugars or polyols. The remaining 4/15 centres (26.7%) strictly prohibited all foods containing natural flavourings due to the potential risk of unlisted or hidden carriers. Dietary guidance concerning cereal-based products and whole grains was highly fragmented. One-third of the participating centres (5/15, 33.3%) reported providing no specific recommendations or restrictions for this food group. Among the remaining 10 centres, clinical approaches varied significantly. Seven centres (7/15, 46.7%) recommended a general preference for refined cereals over whole grains, although they did not mandate the complete exclusion of whole-grain products. One centre (1/15, 6.7%) specifically advised the consumption of refined grains without the germ (e.g., pearled or dehulled grains), while another centre (1/15, 6.7%) recommended avoiding any whole-grain products or bakery items containing more than 3 g of dietary fibre per 100 g of product. Only one institution (1/15, 6.7%) used a highly detailed exclusion list for cereal-based foods. This protocol strictly prohibited pearled whole grains, wholemeal pasta, whole-grain flours, bran, wheat germ, whole-grain cereal creams, tapioca cream, and specific commercial breakfast or bakery products.

### 3.5. Personalization vs. Standardization of Dietary Protocols

Finally, the survey explored the overarching clinical approach regarding long-term dietary prescription. The balance between individualised and standardised protocols was almost equally distributed across centres: 8/15 centres (53.3%) reported that their dietary recommendations were individualised and progressively adjusted based on the patient’s clinical course, metabolic tolerance, and biochemical follow-up. Conversely, the remaining 7/15 centres (46.7%) applied standardised dietary protocols uniformly to all diagnosed patients, regardless of individual clinical phenotypes.

### 3.6. Dietary Supplementation

Due to the strict exclusion of core food groups such as fruits and vegetables, routine micronutrient supplementation represents a critical component of long-term HFI management. Vitamin C (ascorbic acid) was the most frequently prescribed micronutrient, used by nearly all responding centres (15/15, 100%). This was followed by folic acid, which was routinely supplemented in 13/15 (86.6%). In contrast, other supportive supplementations were less commonly integrated into standard care. Docosahexaenoic acid (DHA) and generic multivitamin supplementation were each used by in 5/15 centres (33.3%). Iron supplements were rarely employed, with this intervention reported by only a single institution (6.7%).

### 3.7. Fibre Supplementation and Constipation Management

Chronic constipation is a common clinical challenge in HFI due to restricted dietary fibre intake. However, the strategies used to address this condition varied widely, and one-third of the participating centres (5/15, 33.3%) reported having no clinical experience with fibre supplementation for these patients. Among the remaining 10 centres that manage constipation through specific protocols, approaches were highly heterogeneous. One centre (6.7%) did not specify the type of fibre used, another selected the fibre supplement based strictly on an individual review of the raw ingredient composition, and one centre reported that fibre-related prescriptions were determined solely through multidisciplinary collaboration with the metabolic physician. Pharmacological osmotic laxatives, such as polyethylene glycol (PEG, macrogol), were prescribed in 2/15 centres (13.3%). Targeted fibre supplementation was reported by a single centre (representing 1/15 centres, 6.7%), including insoluble fibre with an ultra-low sugar content of less than 0.1 g per 100 g of product; soluble dietary fibre, specifically polydextrose; partially hydrolysed guar gum (PHGG) verified to be completely free from prohibited sugars or sweeteners; and psyllium husk, used either as a monotherapy or in combination with glucomannan.

**Figure 2:**
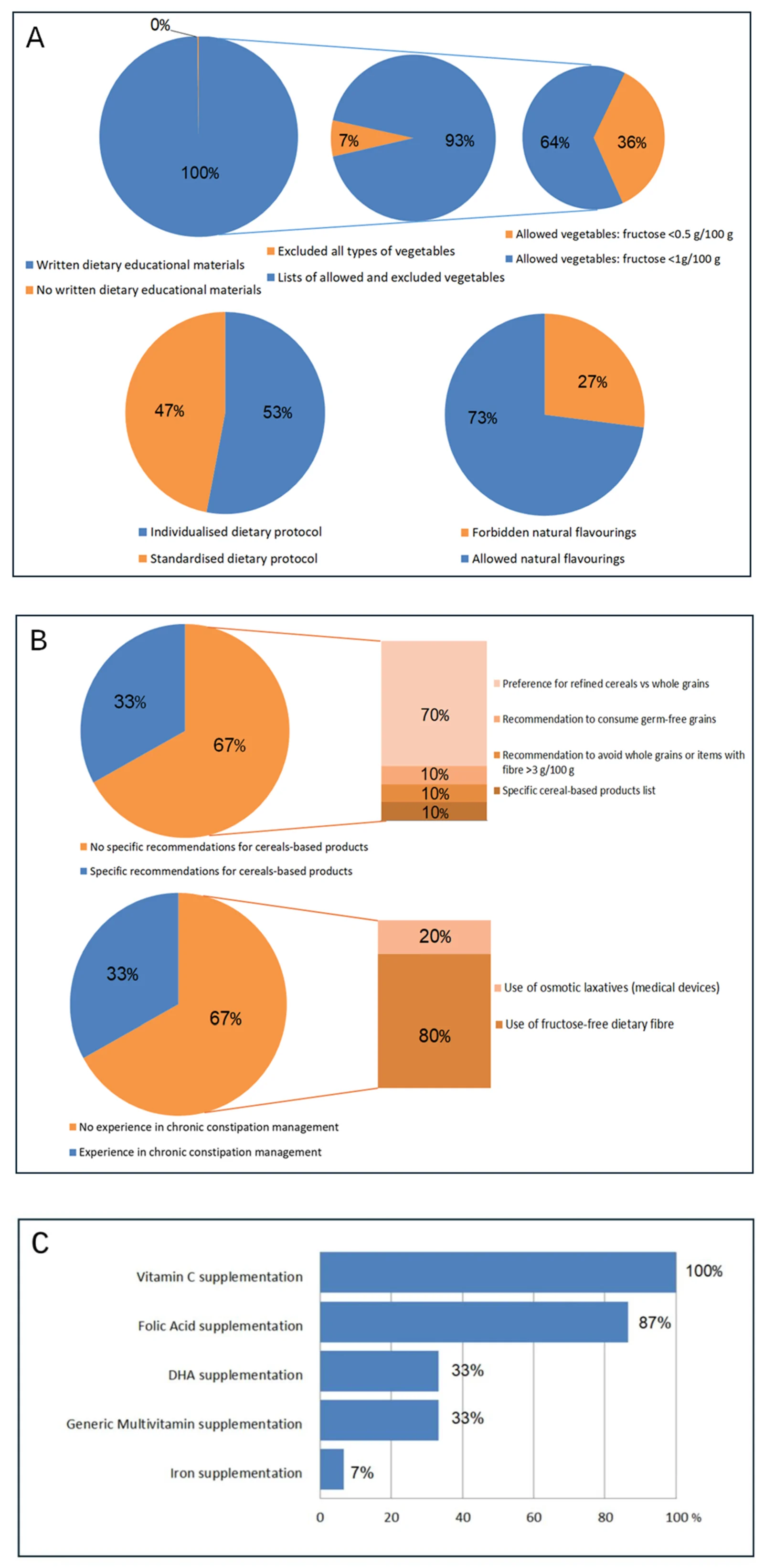
Clinical and dietary management of hereditary fructose intolerance (HFI) among 15 Italian metabolic centres. (**A**) General dietary protocols, approaches to selective vegetable introduction, and regulations regarding natural flavourings. (**B**) Specific recommendations for cereal-based products and therapeutic management of chronic constipation. (**C**) Routine dietary supplementation practices, including micronutrients and DHA. Data are expressed as percentages. Abbreviations: DHA, docosahexaenoic acid.

Overall, variability in dietary management approaches was observed across several domains, including food restrictions, carbohydrate classification, and individualised versus standardised dietary protocols, supplementations and constipation management (Figure 2).

## 4. Discussion

This study represents the first multicentre survey investigating real-world dietary management practices for HFI in Italy. A key demographic finding is that over half of the responding centres provide mixed care for both paediatric and adult patients. This finding suggests that many Italian centres provide care across the paediatric-to-adult age spectrum. Although the availability and organisation of formal transition pathways in Italy were not assessed in this survey, structured transition processes represent an important aspect of long-term care in inherited metabolic disorders, as previously highlighted in the literature [12]. While this operational continuity ensures that complex nutritional expertise is retained within the same clinical team, it highlights an urgent need for standardised transition pathways at a national level. Adult HFI patients often remain under the care of paediatricians or mixed-service dietitians, which may represent a suboptimal arrangement for addressing age-specific metabolic changes and quality-of-life adjustments in adulthood.

Our findings reveal a reassuring consensus regarding foundational patient education, with 100% of the centres providing dedicated instructional materials. However, operationalising these guidelines immediately reveals national fragmentation. This heterogeneity may be related to the absence of specific guidelines and the limited evidence base available to support dietary decision-making in HFI. At present, the tolerable fructose intake in patients with HFI has not been clearly established. However, an intake below 20–40 mg fructose/kg/day (up to approximately 1–2 g/day) is generally considered acceptable in clinical practice [13,14]. In this context, differences in the fructose thresholds used by centres to classify foods as acceptable may contribute to variability in dietary management. In our survey, 35.7% of centres adopted a stricter threshold of <0.5 g fructose/100 g raw product, whereas 64.3% used a threshold of <1 g/100 g. Although a stricter threshold may theoretically further reduce fructose exposure, it may also limit dietary variety and increase the burden of dietary restriction. Conversely, a less restrictive threshold may allow greater dietary flexibility; however, whether these different approaches result in clinically meaningful differences in metabolic control, nutritional status, or long-term outcomes remains unknown. Despite strict dietary restriction, disease-related complications, including hepatic steatosis, may still persist in a substantial proportion of patients [6].

Dietary management is further complicated by the limited availability and reliability of data on fructose content in national food composition databases [15,16], as well as by the lack of sensitive and rapid biomarkers that can be easily integrated with instrumental evaluations and liver and kidney function assessments [9]. Carbohydrate-deficient transferrin (S-CDT%) has been proposed as a potential marker of metabolic control and has shown a correlation with fructose intake; however, its reliability as an indicator of treatment adequacy remains to be fully established [6]. Future investigations may further explore its potential role in tailoring dietary interventions and evaluating interindividual variability in HFI [17].

Despite these limitations, 57.1% of the centres reported allowing the introduction of controlled portions of low-fructose vegetables under conditions of metabolic stability, often with defined serving sizes. However, the survey did not assess whether the food composition databases used to define permitted vegetable lists were consistent across centres. Complete or partial exclusion of vegetables is likely to be one of the factors with the greatest impact on total dietary fructose intake.

In addition, the questionnaire explored attitudes toward foods containing natural flavourings. Notably, 26.7% of the respondents reported avoiding these products as a precautionary measure. Currently, the contribution of sugars from such additives is difficult to quantify objectively, and further studies are warranted. The impact of these restrictions on patient burden also remains unclear. Similar concerns were raised regarding other ingredients, such as polyols and sugars other than fructose. The heterogeneous classification of these compounds across centres may have relevant implications for dietary management. In the absence of clinical studies directly evaluating their safety in individuals with HFI, recommendations are often based on biochemical properties, theoretical metabolic pathways, and precautionary principles. Consequently, differences in classification may influence the extent of dietary restriction, food choices, and the perceived complexity of dietary management, although the impact of these variations on metabolic outcomes, nutritional status, and quality of life remains unknown.

**Figure 3:**
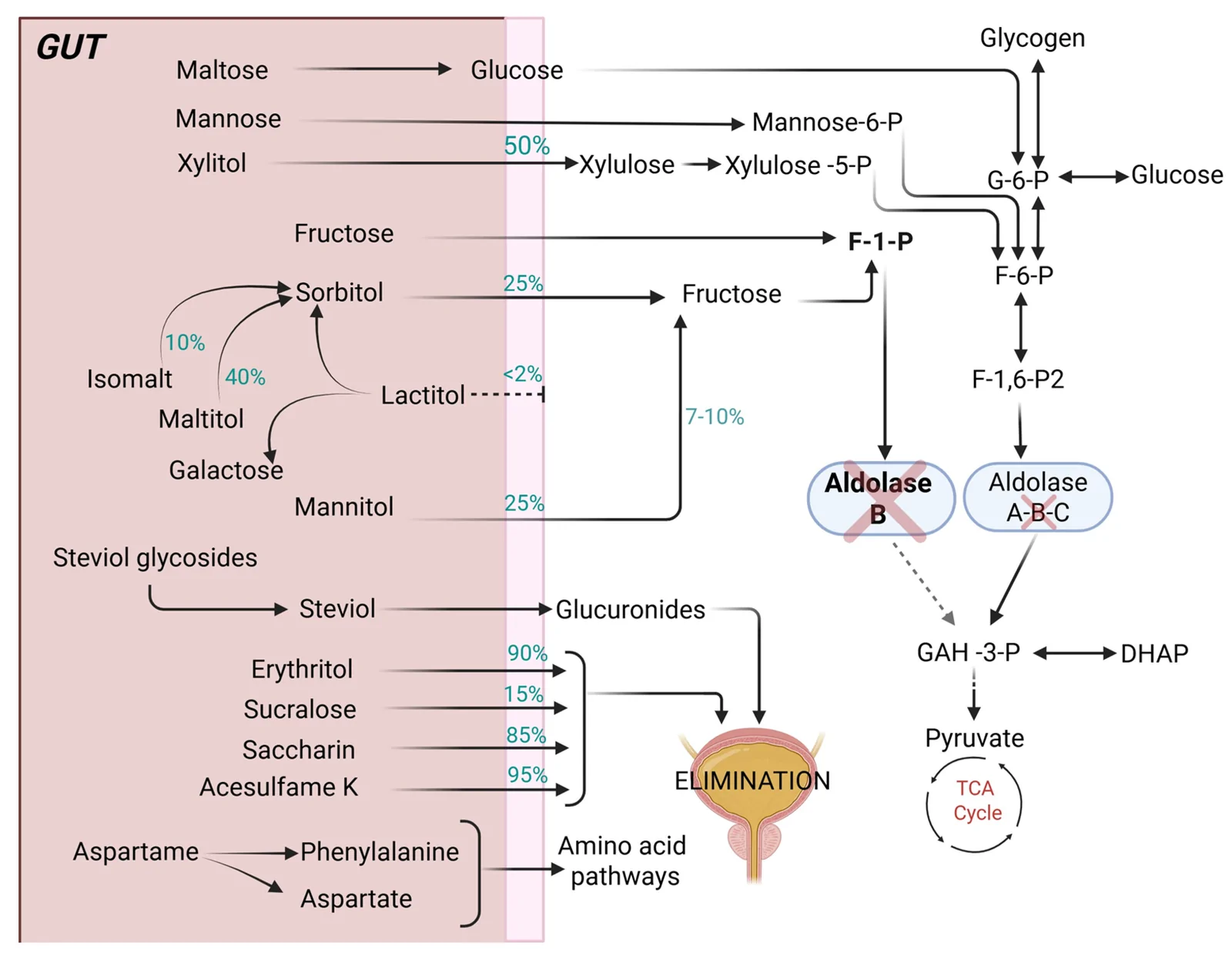
Gastrointestinal absorption, metabolic pathways, and elimination routes of alternative carbohydrates, polyols, and intense sweeteners in relation to the metabolic block in Hereditary Fructose Intolerance (HFI). Percentages indicate estimated intestinal absorption or specific hepatic conversion rates based on current literature. The red “X” denotes the catalytic deficiency of aldolase B, leading to fructose-1-phosphate (F-1-P) accumulation. Abbreviations: DHAP: dihydroxyacetone phosphate; F-1,6-P2: fructose-1,6-bisphosphate; F-6-P: fructose-6-phosphate; G-6-P. glucose-6-phosphate; GAH-3-P: glyceraldehyde-3-phosphate; TCA: tricarboxylic acid.

Several polyols showed consistent restriction patterns across centres, particularly sorbitol (C_6_H_14_O), which was classified as prohibited in all participating centres (15/15, 100%). This consensus is consistent with its known metabolic relevance, as sorbitol can be converted into fructose via sorbitol dehydrogenase, directly contributing to F-1-P accumulation, although only modest amounts are absorbed in the intestine (approximately 25%) [1] (Figure 3). It is important to note that, for many sweeteners and alternative carbohydrates, available information is derived primarily from biochemical pathways, absorption characteristics, and theoretical metabolic considerations rather than from clinical studies performed in individuals with HFI. Therefore, differences in dietary recommendations may reflect varying interpretations of potential metabolic risk and precautionary clinical practice rather than differences in demonstrated clinical outcomes.

Both maltitol (C_12_H_24_O_11_) and isomalt (C_12_H_24_O_11_) are prohibited by the vast majority of centres, with rates of 100% (15/15) and 93% (14/15), respectively. These polyols can be partially hydrolysed in the intestine (approximately 40% for maltitol and 10% for isomalt), releasing sorbitol (supp 1). A variable fraction of this released sorbitol may subsequently be absorbed and metabolised into fructose [13,18]. Although orally administered mannitol (C_6_H_14_O_6_) is absorbed only at approximately 25%, it enters the portal circulation and reaches the liver (Figure 1). Within hepatocytes, it can be converted into fructose by the cytosolic enzyme sorbitol dehydrogenase. Due to a lower binding affinity of the enzyme for mannitol compared to its preferred substrate (sorbitol), this pathway exhibits reduced efficiency, with an estimated conversion rate between 7% and 10% [18] (Figure 3). This free fructose is subsequently phosphorylated by fructokinase into F-1-P. This potential metabolic pathway may contribute to the cautious approach adopted by several centres, although its clinical relevance in HFI remains incompletely defined. Lactitol (C_12_H_24_O_11_) and xylitol (C_5_H_12_O) showed intermediate and more heterogeneous classifications, with a substantial proportion of centres allowing their use under specific conditions, despite limited and indirect metabolic links to fructose metabolism. Lactitol is a polyol that has been shown to be absorbed in an amount of less than 2% (Figure 3). The majority of it is hydrolysed in the intestine, producing galactose and sorbitol; the latter could then be converted into fructose, although the resulting dose is likely metabolically negligible [18]. Therefore, lactitol may explain the cautious classification adopted by some centres despite its limited contribution to fructose metabolism. Xylitol is a polyol with an intestinal absorption rate of approximately 50% [18]. Although it was prohibited in 46.7% of Italian centres, xylitol can be partially metabolised to xylulose-5-phosphate and subsequently to intermediates of the pentose phosphate pathway, without involving the metabolism of F-1-P [19]. Despite the lack of clinical studies demonstrating their safety in this patient population, xylitol does not appear to cause of F-1-P accumulation. In contrast, erythritol (C_4_H_10_O_4_) and steviol glycosides (C_38_H_60_O_18_) demonstrated the highest variability in dietary recommendations. Erythritol was generally considered acceptable by most centres, likely reflecting its minimal involvement in carbohydrate metabolism and extensive urinary excretion. However, clinical safety data specifically in HFI patients are lacking [18]. A minority of centres still reported restrictions, highlighting uncertainty in clinical practice. It is largely absorbed in the small intestine (up to 90%) and appears not to be metabolised by the host, with the fraction that escapes absorption also showing no evidence of fermentation by the gut microbiota [20]. Steviol glycosides showed similar heterogeneity, likely influenced more by concerns regarding product composition and potential contamination with mono- or disaccharides than by intrinsic metabolic pathways. Steviol glycosides undergo microbial hydrolysis in the colon to yield steviol, which is absorbed and subsequently metabolised to steviol glucuronide, primarily excreted in the urine in humans [21] (Figure 3). However, this conclusion is based on biochemical characteristics rather than clinical studies in individuals with HFI, and the long-term effects of regular consumption in this population remain unknown. However, concerns may arise regarding the purity of commercially available sweeteners and the potential presence of bound monosaccharides such as glucose, rhamnose, xylose, galactose, arabinose, and fructose. Artificial sweeteners such as aspartame, saccharin, and acesulfame K were predominantly considered safe, with high rates of permissive classification (80–93%). Sucralose (C_12_H_19_C_l3_O_8_) also showed mixed recommendations across centres, despite evidence of minimal metabolic involvement and negligible participation in carbohydrate pathways. Sucralose is a disaccharide derived from sucrose through a chemical process in which three hydroxyl groups are replaced by chlorine atoms [22]. It is classified as a non-nutritive artificial sweetener because, although approximately 15% of ingested sucralose is absorbed at the intestinal level, it is subsequently excreted unchanged in the urine and does not appear to participate in carbohydrate metabolic pathways based on available biochemical and pharmacokinetic data [23]. Nevertheless, direct evidence regarding sucralose consumption in HFI patients is unavailable. Sweeteners such as sucralose and steviol glycosides exhibit a very high sweetening power, up to approximately 600-fold and 200-fold greater than that of sucrose, respectively [23,24]. As a result, only very small amounts are required to achieve a palatable taste in food products. Furthermore, for sucralose, strict regulatory limits are established in the European Union, including a maximum of 40 mg/100 g in light yogurts, 30 mg/100 mL in flavoured beverages, and 20 mg/100 g in flour-based snacks [25]. Mannose (C_6_H_12_O_6_) and maltose (C_12_H_22_O_11_) showed inconsistent classifications despite their limited or indirect relevance to fructose metabolism, suggesting a precautionary approach in some centres. Mannose, which was reported as prohibited in 20.0% of centres, is a monosaccharide found in small quantities in fruits and vegetables. It is also a component of many additives like gellan gum (E418), guar gum, and xanthan gum. Mannose is metabolised in humans by mannose phosphate isomerase into F-6-P, entering the pathway of gluconeogenesis–glycolysis and can thus be metabolised to glucose [26] (Figure 3). Maltose is a disaccharide composed of two glucose molecules, so it should not raise many concerns as it does not contain fructose and would not be expected to directly contribute to F-1-P accumulation [27] (Figure 1). Glucose syrup, polydextrose (C_6_H_10_O_5_), sodium cyclamate (C_6_H_12_NNaO_3_S), and ammonia sulphite caramel (E150d) exhibited further heterogeneity in dietary recommendations, reflecting differences in both their chemical composition and metabolic relevance to fructose metabolism. Glucose syrup, primarily composed of glucose polymers of variable chain length, is generally considered of limited metabolic concern in the context of HFI, as it does not contain fructose and is metabolised via glycolytic pathways without contributing to F-1-P accumulation [13]. However, its degree of hydrolysis and variable composition may lead to small amounts of free fructose and, in some preparations, trace saccharides that could explain occasional precautionary restrictions in clinical practice. Furthermore, European Regulations (Council Directive 2001/111/EC of 20 December 2001 relating to certain sugars intended for human consumption) [28] mandate that the fructose content must remain below 5% (on a dry matter basis) for the product to be labelled as “glucose syrup,” thereby legally permitting the presence of residual trace amounts of fructose. Although a fraction below 5% is generally considered negligible in the dietary management of HFI, vigilance is required for commercial food products where glucose syrup is a primary ingredient. Under these circumstances, tolerability depends heavily on the average portion size intended for consumption by the patient. Polydextrose, a synthetic glucose polymer used as a bulking agent and dietary fibre, is only partially fermented by the gut microbiota and has negligible absorption in the small intestine [29]. Consequently, it is not expected to generate fructose or fructose-derived metabolites, nor to interfere directly with hepatic fructose metabolism. Nevertheless, its fermentation products (short-chain fatty acids) and the variability in commercial formulations may contribute to inconsistent classification across centres. Sodium cyclamate is largely excreted unchanged in humans, with a portion potentially converted by intestinal microbiota into cyclohexylamine [30]. Importantly, neither compound enters carbohydrate metabolic pathways, and no mechanistic link to fructose metabolism or F-1-P accumulation has been described. Ammonia sulphite caramel is produced through controlled heat treatment of carbohydrates (often glucose or sucrose) in the presence of ammonium and sulphite compounds. Although it may originate from carbohydrate substrates, the final product consists of complex, highly polymerized compounds with no meaningful contribution to monosaccharide absorption [13]. These findings highlight that dietary recommendations for sweeteners in HFI are not solely driven by metabolic evidence, but also by precautionary principles and variable interpretation of biochemical pathways.

Vitamin supplementation practices showed a relatively more homogeneous pattern across centres, with vitamin C and folic acid being the most commonly prescribed supplements, reflecting concerns regarding potential deficiencies associated with the avoidance of fruits and vegetables [31]. This likely reflects a precautionary approach in the context of restrictive dietary regimens, aimed at preventing potential micronutrient deficiencies, although robust evidence supporting routine supplementation in HFI remains limited [31,32], particularly given the lack of clinical evidence or biochemical assessments demonstrating actual nutritional deficiencies in these patients. Regarding bone metabolism, deficiencies in vitamin D have been reported in the literature [1]; however, clinical evidence suggests this is not a primary diet-related deficiency, but rather a secondary complication occurring exclusively in patients with compromised renal function, where the renal conversion of vitamin D to its active form is impaired [1]. Five centres reported the use of DHA supplementation. This is of interest, as DHA intake is unlikely to be significantly affected by the dietary restriction in HFI [33]; however, its potential role in liver disease has been investigated, with limited evidence suggesting possible benefits in both alcoholic and non-alcoholic fatty liver disease [34,35,36].

In contrast, chronic constipation management strategies were highly heterogeneous, both in terms of the nutritional supplements and medical devices used and the clinical indications for their use. Approaches ranged from no clinical experience to the use of osmotic laxatives such as polyethylene glycol, as well as various fibre preparations including psyllium, partially hydrolysed guar gum, and other soluble or insoluble products, often selected based on individual clinician preference rather than standardised protocols. This variability may reflect the absence of specific dietary guidelines for gastrointestinal symptom management in HFI, particularly for constipation, and the uncertainty regarding the interaction between fibre composition and residual sugar content. Similarly, the lack of specific recommendations for cereal-based products reported by some centres highlights another area of uncertainty. Although cereal products may represent an important source of dietary fibre and contribute to gastrointestinal health, their suitability in HFI requires careful evaluation of carbohydrate composition and the potential presence of prohibited sugars or sweetening agents. Identifying safe fibre-containing foods is therefore essential to balance strict fructose avoidance with the prevention of long-term gastrointestinal complications. Crucially, this operational uncertainty highlights a critical safety gap regarding the choice of commercial fibre supplements. Many products formulated for constipation contain inulin, a fructan polymer composed of fructose chains. Despite being classified as a non-digestible carbohydrate, inulin is composed entirely of fructose residues and therefore warrants consideration in fructose-restricted conditions such as HFI [37]. However, the clinical relevance of this potential exposure in HFI patients has not been established. The fact that dietary protocols must strictly screen not only for free fructose, sucrose, and sorbitol but also for hidden polymers like inulin in formulations for constipation underscores the complexity of managing gastrointestinal symptoms in HFI without standardised guidelines. While micronutrient supplementation is relatively standardised, the management of gastrointestinal symptoms through fibre supplementation remains highly inconsistent across centres, potentially contributing to differences in dietary management approaches. Furthermore, the clinical relevance of monitoring real-world dietary patterns lies in their direct impact on gastrointestinal wellbeing. In the general population, highly restrictive exclusion diets or patterns low in dietary fibre are well documented drivers of altered gut motility, chronic constipation, and impaired microbiota homeostasis [38]. In HFI patients, the mandatory, lifelong avoidance of most plant-based foods inherently enforces a selective dietary pattern that predisposes to chronic motility issues. This explains the high heterogeneity observed in our survey regarding constipation management and underscores the challenge of balancing strict fructose avoidance with the clinical necessity of supporting long-term gut health.

Importantly, the present survey was designed to describe variability in centre-level clinical practices and was not intended to define optimal dietary management strategies or generate clinical recommendations. The findings should therefore be interpreted as a description of current practice patterns rather than as evidence supporting one dietary approach over another. This study has several limitations that should be acknowledged. First, the sample size was relatively small and limited to centres within a single national network. However, this reflects the rarity of HFI and the limited number of specialised metabolic centres managing this condition in Italy. Moreover, the study was designed as an exploratory descriptive survey aimed at providing the first overview of current dietary management practices for HFI in Italy, rather than evaluating associations between dietary approaches and clinical outcomes. Second, data were self-reported by dietitians and may therefore reflect reported institutional practices rather than actual patient-level implementation, adherence, or metabolic outcomes. Nevertheless, this approach was considered appropriate to identify current clinical practices in a field where standardised recommendations and published data are lacking. Third, the survey design did not allow for an in-depth exploration of the clinical rationale underlying certain dietary choices, particularly in areas showing high variability, such as permitted vegetables, sweetener classification, and fibre supplementation. In addition, the questionnaire did not specifically distinguish between vegetables and legumes, nor did it collect information regarding cooking methods, both of which may influence dietary management practices. Finally, although responses were not externally validated, the involvement of experienced metabolic dietitians from multiple specialised centres provides valuable insight into current real-world clinical practice.

Recent evidence has improved understanding of real-life dietary management in HFI. In a recent nationwide cohort study by Izquierdo-García et al. [39], 36 patients with HFI were evaluated using dietary records and a food frequency questionnaire. The authors reported that most patients successfully maintained their total fructose intake within the recommended safety limits (9.79 mg/kg/day). Furthermore, the same study highlighted that patients with HFI exhibit a distinctly different dietary pattern to the general population, consuming a significantly lower percentage of carbohydrates (39.6% of total energy intake, TEI) and dietary fiber (12.0 g/day), accompanied by a marked shift toward higher intake of proteins (92.4 g/day vs. 70.4 g/day in healthy controls; 18.0% TEI) and dietary fats (43.7% TEI). This profile reflects a spontaneous macronutrient adaptation to compensate for the strict restriction of fructose-containing foods.

Overall, these findings highlight that while strict theoretical recommendations do not always perfectly align with real-world practices, patients achieve compliance with safety guidelines by consuming high-fructose foods less frequently and in smaller, controlled quantities, underscoring the need for updated data on the fructose content of foods to optimize dietary counselling in HFI. Recent lipidomic evidence has further expanded the understanding of long-term metabolic outcomes in HFI. A study demonstrated that diet-treated HFI patients exhibit a distinct serum lipidomic profile compared with healthy controls, together with a high prevalence of liver steatosis and markers of low-grade systemic inflammation [40]. These findings suggest that, despite strict dietary management, HFI may be associated with previously underrecognized metabolic and cardiovascular risk factors. This emerging evidence highlights the need for a more comprehensive evaluation of long-term outcomes in HFI beyond dietary fructose restriction alone.

## 5. Conclusions

To our knowledge, this study represents the first national survey specifically investigating dietary management practices in individuals with HFI. By collecting real-world data from specialised metabolic dietitians across multiple Italian centres, this investigation provides a comprehensive overview of current clinical approaches in the absence of formal, international consensus guidelines. Our findings highlight marked heterogeneity in dietary recommendations across the country, particularly regarding the tolerance thresholds for vegetables, alternative sweeteners, complex carbohydrates, and fibre supplements. This operational variability directly reflects the scarcity of robust clinical evidence, persistent gaps in food composition databases, and the current lack of validated, dynamic biomarkers for metabolic control. Consequently, current clinical management relies heavily on a precautionary principle rather than evidence-based thresholds. While a cautious approach is highly understandable given the severity of acute fructose-related toxicity, overly restrictive dietary protocols may potentially increase dietary burden and could have implications for nutritional adequacy and long-term dietary adherence, which warrant further investigation. These include a severe reduction in dietary variety, an increased risk of specific micronutrient deficiencies, and a substantial negative impact on patients’ daily quality of life and long-term treatment adherence. Collectively, these findings underscore the urgent need for well-designed, prospective clinical studies to precisely define the physiological tolerance of selected dietary components and additives in HFI. Establishing such evidence is an essential prerequisite to support the development of standardised, international dietary recommendations. Future evidence-based frameworks may help balance metabolic safety with nutritional adequacy and long-term patient-centred care.

## Figures and Tables

**Table 1:** Classification of sugars, polyols, and sweeteners as prohibited, permitted, permitted but in controlled amounts, or unspecified in dietary management of HFI across Italian centres.

Sugars/Sweeteners	Prohibited	Permitted	Permitted but in Controlled Amounts	Unspecified *
Maltitol	*n* = 15/15 (100%)			
Sorbitol	*n* = 15/15 (100%)			
Mannitol	*n* = 14/15 (93.3%)		*n* = 1/15 (6.7%)	
Isomalt	*n* = 14/15 (93.3%)	*n* = 1/15 (6.7%)		
Xylitol	*n* = 7/15 (46.7%)	*n* = 6/15 (40.0%)		*n* = 2/15 (13.3%)
Erythritol	*n* = 3/15 (20.0%)	*n* = 9/15 (60.0%)		*n* = 3/15 (20.0%)
Lactitol	*n* = 1/15 (6.7%)	*n* = 7/15 (46.7%)		*n* = 7/15 (46.7%)
Glucose syrup	*n* = 5/15 (33.3%)	*n* = 8/15 (53.3%)	*n* = 1/15 (6.7%)	*n* = 1/15 (6.7%)
Mannose	*n* = 3/15 (20.0%)	*n* = 5/15 (33.3%)		*n* = 7/15 (46.7%)
Stevia	*n* = 4/15 (26.7%)	*n* = 9/15 (60.0%)		*n* = 2/15 (13.3%)
Maltose	*n* = 2/15 (13.3%)	*n* = 11/15 (73.3%)	*n* = 1/15 (6.7%)	*n* = 1/15 (6.7%)
Polydextrose	*n* = 3/15 (20.0%)	*n* = 6/15 (40.0%)		*n* = 6/15 (40.0%)
Sucralose	*n* = 5/15 (33.3%)	*n* = 3/15 (20.0%)	*n* = 3/15 (20.0%)	*n* = 4/15 (26.7%)
Saccharin		*n* = 14/15 (93.3%)	*n* = 1/15 (6.7%)	
Acesulfame K		*n* = 12/15 (80.0%)		*n* = 3/15 (20.0%)
Sodium cyclamate		*n* = 6/15 (40.0%)		*n* = 9/15 (60.0%)
Aspartame	*n* = 1/15 (6.7%)	*n* = 13/15 (86.7%)	*n* = 1/15 (6.7%)	
Ammonia sulphite caramel (E150d)	*n* = 5/15 (33.3%)	*n* = 2/15 (13.3%)	*n* = 1/15 (6.7%)	*n* = 7/15 (46.7%)

* unspecified: the patient is not given a written indication, so it is neither prohibited nor allowed.

## Data Availability

The original contributions presented in the study are included in the article and Supplementary Materials. Further inquiries can be directed to the corresponding author.

## Supplementary Materials

The following supporting information can be downloaded at: https://www.mdpi.com/article/10.3390/nu18162609/s1, Supplementary Material File S1; Hereditary Fructose Intolerance Questionnaire—Dietary Practices 2026.
