# Supplementary material for: Current Dietary Practices in Hereditary Fructose Intolerance: Results from a National Survey Across 15 Italian Metabolic Centres
Source: Nutrients. 2026 Aug 10;18(16):2609. doi: 10.3390/nu18162609 (PMC13516941; doi:10.3390/nu18162609)
Supplement: Supplementary file 1 [file nutrients-18-02609-s001.zip › nutrients-4473132-supplementary.pdf]

Hereditary Fructose Intolerance Questionnaire – Dietary Practices 2026

1. Are patients with Hereditary Fructose Intolerance (HFI) followed in your centre?
    a. Adults
    b. Pediatric patients
    c. Both
    d. We do not follow patients with HFI

Dietary practices: I

2. Are written dietary guidelines provided, including lists of prohibited and permitted foods?
    a. Yes
    b. No

3. Vegetables are:
    a. All excluded
    b. Divided into: excluded and included

Dietary practices: II

4. If some vegetables are allowed: what is the maximum fructose content per 100g considered acceptable for inclusion?
    a. <0.5g/100g
    b. <1g/100g
    c. Other

5. For allowed vegetables, are indicative maximum portion sizes provided?
    a. Yes
    b. No

Dietary practices: III

Please indicate whether the following sugars/sweeteners are prohibited, permitted, or not specified in your HFI local dietary recommendations:

Sugars/sweeteners	Prohibited	Permitted	Permitted but in controlled amounts	Unspecified
6. Maltitol				

7. Sorbitol				
8. Mannitol				
9. Isomalt				
10. Xylitol				
11. Erythritol				
12. Lactitol				
13. Glucose syrup				
14. Mannose				
15. Stevia				
16. Maltose				
17. Polydextrose				
18. Sucralose				
19. Saccharin				
20. Acesulfame K				
21. Sodium cyclamate				
22. Aspartame				
23. Ammonia sulphite caramel (E150d), e.g. Coca Zero coloring				

24. Are foods containing natural flavourings permitted (if they do not contain other prohibited sugars)?
    a. Yes
    b. No

25. Are guidelines also provided regarding cereal-based products? (e.g., whole grain vs refined). If yes, please briefly describe them.

26. Are dietary recommendations individualised?
    a. No, they are standard for all patients
    b. Yes, they are adjusted according to clinical progress

27. Are dietary supplements normally prescribed? (multiple choice)
    a. Vitamin C
    b. Folic Acid
    c. DHA
    d. Multivitamin
    e. Other (please describe):

28. What type of fiber supplements are used (in cases of constipation)?
